# Social and Behavioural Outcomes of School Aged Autistic Children Who Received Community-Based Early Interventions

**DOI:** 10.1007/s10803-022-05477-3

**Published:** 2022-02-19

**Authors:** Zoe Vinen, Megan Clark, Cheryl Dissanayake

**Affiliations:** 1 Olga Tennison Autism Research Centre, La Trobe University, Melbourne, Australia; 2 Autism Specific Early Learning and Care Centre, La Trobe University Melbourne, Bundoora, Melbourne, VIC 3086 Australia

**Keywords:** School age, G-ESDM, Social, Behavioural, Peer play, Adaptive outcomes

## Abstract

The school-age outcomes of autistic children who received early interventions (EI) remains limited. Adaptive functioning, social, peer play skills, problem behaviours, and attitudes towards school of 31 autistic children who received community-based group early start Denver model (G-ESDM) were compared to 28 age matched autistic children who received other community interventions. Similar adaptive behaviours, social skills, problem behaviours and attitudes towards school were found. Play disruption was the only dimension of play to differ; children that received community interventions demonstrated higher levels of play disruption compared to the G-ESDM group. Children had pervasive challenges in adaptive behaviour, social and play behaviour at school, irrespective of EI type. Thus, ongoing provisions are needed to support development into the school years.

## Introduction

The benefits of early intervention (EI) for children with a diagnosis of autism spectrum disorder (ASD; hereafter autism) are well-established, with improvements in language, cognition, and adaptive behaviour widely documented (Reichow, 2012; Rogers et al., 2019; Smith & Iadarola, 2015; Warren et al., 2011). Yet, to date there has been little examination of outcomes following the cessation of EI, with the developmental trajectory and later outcomes, particularly during the school years, poorly understood.

As EI assists children to engage with and learn from their environment, social and otherwise, it is expected that autistic children in receipt of EI would continue learning and developing following EI cessation. Despite known difficulties with generalisation (Church et al., 2015; de Marchena et al., 2015; Hwang & Hughes, 2000), a recent review found that 8 of the 9 randomised control trials (RCTs) reported gains in learning amongst autistic children both within the intervention context, and generalisation across people, setting and/or activity. Importantly, generalisation varied across studies and skills, which is unsurprising given the heterogeneity in profiles of autistic children (Fountain et al., 2012; Jeste & Geschwind, 2014) that likely impact the rate of acquisition in response to EI, and the extent of generalisation. Learning skills within a naturalistic setting (i.e., at day-care, home and school) facilitates generalisation (Schreibman et al., 2015), which underpins the foundation of Naturalistic Developmental Behavioural Interventions (NDBIs) delivered in group environments. These interventions may therefore promote greater generalisation of skills into other social settings, such as the school playground.

School is the first opportunity whereby autistic children apply the skills learnt during EI in a new and socially complex setting, amongst their peers. Autistic students tend to experience greater behavioural and emotional difficulties, including aggressive and oppositional behaviour, anxiety, depression, attentional difficulties and hyperactivity relative to their typically developing (TD) peers (Ashburner et al., 2010; Macintosh & Dissanayake, 2006). Consequently, they are more likely to be on the periphery of social activity (Chamberlain et al., 2007), have fewer and poorer quality friendships (Bauminger & Kasari, 2000; Macintosh & Dissanayake, 2006), and experience increased loneliness (Bauminger & Kasari, 2000). However, social outcomes, while imperative for school adjustment, are often considered secondary to outcomes relating to academic success, such as language and cognition.

School-age follow-up studies yield important information on learning beyond the EI years. Starr et al. (2016) found many school-aged autistic children progressed beyond the gains made during intervention, while others regressed, experiencing pervasive social and behavioural difficulties at school. Clark et al. (2018) found earlier autism diagnoses (by 2-years) and commencement of EI at a younger age, contributed to improved cognition and language at school, while autism symptoms remained stable. Indeed, many EI outcome studies report developmental gains amongst autistic children post intervention, with autism symptomatology remaining stable over time (Fountain et al., 2012; Vivanti et al., 2014, 2016).

While autism symptoms, cognition and language remain the most common measures of outcome at school age, Towle et al. (2014) concluded that outcomes should be broadened beyond symptomatology and cognition, to capture more functional abilities such as adaptive behaviour. The limited research into adaptive functioning at school age has returned mixed results, with both improvements (Akshoomoff et al., 2010; Landa & Kalb, 2012; Magiati et al., 2011), and enduring adaptive functioning difficulties reported into the school years (O’Connor & Healy, 2010).

Other relevant school related behaviours such as social and peer interaction skills and attitudes towards school remain under researched. O’Connor and Healy (2010) highlighted the behavioural (inattention, hyperactivity) and social difficulties, including anxiety, amongst five school-aged autistic children. Similarly, social difficulties were found by Kasari et al. (2011) when comparing the social networks and friendships of 120 cognitively able children with (*n* = 60) and without Autism (*n* = 60). Significant differences in the friendships between groups were identified, such that autistic children nominated fewer peers as friends and received fewer friendship nominations from classmates. Importantly, while autistic children had lower friendship quality, fewer peer connections and reciprocal friendships overall, a proportion of them (20%) *were* identified as having reciprocal friendships. Peer engagement was the most distinguishing variable between groups—autistic children were less socially engaged than their classmates; even those with a reciprocal friendship were not engaged on the playground.

Peer engagement difficulties were also identified in a recent study of social and peer play skills (play interaction, play disruption and play disconnection) and problem behaviours amongst 35 autistic children aged 7 to 9 years (Clark et al., 2019). Parents and teachers alike reported similar social skills at home and school, while problem behaviours were highest at home. Informants emphasised similar peer engagement difficulties across settings, whereby play disconnection was the most problematic, indicating that autistic children had difficulties engaging in play, even when invited to do so by peers or siblings. Given the social difficulties experienced by autistic children, it is unsurprising that many seek adult interaction for play rather than peers and engage mostly in solitary functional play (Holmes & Willoughby, 2009), with increased repetitive behaviours linked with less engagement in play (Honey et al., 2007). Despite the importance of social skills for assimilation into the school environment, the knowledge of play behaviours of school-aged autistic children following EI remains limited.

Positive perceptions by children of their school environment are known to promote healthy development and enhance school performance (Van Ryzin, 2011). However, the attitudes of autistic children towards school in the early school years—when such attitudes are forming—remain largely unknown. Understanding attitudes towards school is important, especially given the high rates of school refusal amongst this population of children (Bitsika et al., 2021; McClemont et al., 2020; Munkhaugen et al., 2017; Ochi et al., 2020).

### The Early Start Denver Model

The ESDM is a NDBI for young autistic children aged 12 to 48 months (Dawson et al., 2010; Rogers, 2013), that embeds learning into naturally occurring activities such as mealtimes and play to facilitate social engagement. A RCT established the efficacy of the ESDM documenting significant improvements in cognition and adaptive behaviour amongst young autistic children following two-years of 1:1 EI compared to a community treatment-as-usual control group (Dawson et al., 2010). This same cohort were followed-up two years post EI cessation (M_age_ 6-years) where improved adaptive behaviour and fewer autism symptoms were found in the ESDM group, with equivalent gains in cognition and language evident in both groups (Estes et al., 2015). Others have reported similar improvements in cognition, adaptive behaviour and social communication in autistic children who received community-based group ESDM (G-ESDM; Eapen et al., 2013; Vivanti et al., 2013, 2014, 2016). A recent meta-analysis of 12 ESDM studies between 2010 and 2019 (Fuller et al., 2020) followed children from pre-school to school age (6 to 9 years) with consistent improvements in language and cognition identified. In contrast, the adaptive behaviour and social communication outcomes were variable across studies. Collectively, these findings emphasise the positive short-term outcomes for autistic children following ESDM cessation, with preliminary evidence of sustained developmental improvements extending beyond the EI period, into the early schoolyears.

Unlike the traditional ESDM, G-ESDM targets learning objectives such as engagement and intentional communication within small group activities with a child-staff ratio of 1:3. The G-ESDM has been deemed feasible and effective when delivered within a childcare setting (Vivanti et al., 2014), and has since been manualized (Vivanti et al., 2017). The first study to report on the school-age outcomes of autistic children aged 6 to 9 years following receipt of G-ESDM was conducted by Vinen et al. (2018). The outcomes of the G-ESDM cohort were compared to children that received other community intervention provisions. Significant improvements in cognition and social affect were evident in both groups at follow-up, irrespective of EI type, while autism symptoms, particularly restricted and repetitive behaviours, increased by school age. While this study found no differences in cognition or behaviour between groups, other important and functional outcomes necessary for adjustment to the school environment are yet to be explored. To that end, with only limited accounts of long-term trajectories following G-ESDM cessation, further work is needed to establish the social and behavioural developmental trajectories post G-ESDM intervention, particularly into the school years.

### Aims and Hypotheses

The current study extends the work of Vinen et al. (2018) by examining other school-related outcomes including social and peer play skills, problem behaviours, adaptive functioning, and attitudes towards school in a cohort of school-aged autistic children that received G-ESDM within their community day care setting in their pre-school years. Their outcomes were compared to an age-matched cohort of children that received standard community EI.

Given the mixed adaptive behaviour outcomes in response to the ESDM (Fuller et al., 2020), and enduring adaptive functioning difficulties reported elsewhere (O’Connor & Healy, 2010), adaptive functioning was not expected to distinguish groups at school age. As the ESDM targets social attention, imitation, language, play skills, affect sharing and social orientation (Rogers et al., 2012), children in receipt of G-ESDM were expected to display increased social skills and better peer play behaviours relative to children receiving standard community interventions. Further to this, the long-term reduction in challenging behaviour found by Estes et al. (2015) in response to the ESDM, and reduced maladaptive behaviours reported by Fulton et al., (2014), children in receipt of G-ESDM were expected to exhibit fewer problem behaviours at follow-up relative to children in receipt of other community interventions. No hypotheses were proposed regarding differences in children’s school liking and avoidance, as this has yet to be explored in school-aged autistic children.

## Method

### Participants

The current study reports on the same cohort of school-aged autistic children (aged 6–9 years) as studied by Vinen et al. (2018). All had a confirmed community-based diagnosis of ASD (Diagnostic and Statistical Manual of Mental Disorders; American Psychiatric Association, 2000). Importantly, both groups were matched on chronological age, autism severity and cognition at entry into intervention (see Table 1 for details of participant characteristics).

**Table 1:** Participant characteristics at baseline

Time 1—baseline	ESDM *N* = 31	Comparison *N* = 28	t-Test p-value
Gender, M, F	27, 4	25, 3	–
Chronological age T1 (months): *M* (SD)	39.16 (9.91)	35.46 (7.62)	.12
T1 ADOS, calibrated severity score: *M* (SD)	7.39 (2.09)	6.68 (2.20)	.21
T1 MSEL, verbal DQ: *M* (SD)	50.61 (23.27)	48.14 (22.66)	.68
T1 MSEL, nonverbal DQ: *M* (SD)	64.80 (20.80)	68.88 (17.38)	.42

*ESDM* early start Denver model, *M* male, *F* female, *T1* time 1, *ADOS* autism diagnostic observation schedule, *MSEL* Mullen scales of early learning, *DQ* developmental quotient

#### G-ESDM Cohort

The families of 41 children that had attended the G-ESDM intervention program at the Victorian Autism Specific Early Learning and Care Centre (ASELCC) and were aged between 6 and 9 years were invited to participate in the current study. Thirty-one families responded to the invitation.

#### Comparison Cohort

Children from the community with a confirmed diagnosis on the autism spectrum, aged between 6 and 9 years in receipt of other community interventions were eligible to participate in the study. Families affiliated with the Child Development Unit at the Olga Tennison Autism Research Centre, whom had agreed to be contacted for future research, were invited via the participant registry. Families in the wider community responded to a study advertisement on social media. No specific exclusion criteria were applied to this study. Twenty-eight participants that had received a standard intervention program comprised the comparison group (M_age_ = 7 years, *SD* = 11.05).

#### Family Demographics

Families in both groups were predominantly Caucasian (61.3% ESDM and 50.3% comparison) or Asian (30.2% ESDM and 40.2% comparison) middle class families with the mean annual income in both groups AUD$75–$95K. Working arrangements were similar across groups with 40% of parents in each group in the workforce. However, caring responsibilities differed by group with more parents in the ESDM group nominating caring and home duties as their primary role relative to the comparison group (43% vs 27% respectively).

### Early Start Denver Model

Group ESDM (G-ESDM; Vivanti et al., 2017) utilizes the ESDM principles and strategies to target individual learning objectives within a group childcare setting. Trained therapists use a semi-structured assessment protocol (the ESDM curriculum checklist) to identify each child’s learning objectives across multiple developmental domains. Goals are then integrated within naturalistic play-based routines, providing opportunities for interactions between children and staff (e.g., book activities or song routines). Groups typically comprise three to four children with a trained therapist. Objects that draw upon the children’s interests are used to enhance engagement in play, verbal and nonverbal communication, gestural and vocal imitation, and social engagement (including giving and sharing objects, turn-taking and joint attention). Activities relevant to cognitive goals (i.e., matching or counting) and social goals (i.e., initiating interactions and play skills) are also implemented into the program.

Therapists in the ESDM program included early education teachers, childcare workers, and allied health professionals (speech pathologists, psychologists, and occupational therapists), who work as a part of a transdisciplinary team. To ensure the ESDM was implemented in the way it was originally intended, therapists adhered to a multi-step fidelity process that assesses 13 therapist behaviours on a 5-point Likert Scale (see Rogers & Dawson 2010 for further details on fidelity). The staff:child ratio varies from 1:3 to 1:4 based on the time of day, activity and ability level of the child. Six two-hour information sessions on the ESDM were held for parents who were encouraged to implement ESDM strategies at home.

### Community-Based Intervention

Standard community-based intervention strategies can include 1:1 EI or group-based interventions. These typically utilize visual supports (i.e., visual schedules), TEACCH program strategies (Treatment and Education of Autistic and related Communication Handicapped Children (TEACCH; Schopler, 1994), and Applied Behaviour Analysis (ABA) strategies (i.e., pivotal response training, naturalistic teaching strategies; see National Autism Centre, 2009). When indicated, augmentative communication systems (i.e., Picture Exchange Communication System PECS; Frost & Bondy, 1994) are also used. Structured large and small group activities provide learning opportunities, with further teaching opportunities incorporated throughout the day during free play time, snack, outside play, and self-help activities. Speech and occupational therapy are also sought both within services or by external providers.

Classrooms are run by an early years or special education teacher and supported by a larger multidisciplinary team, including speech pathologists, occupational therapists, early years teachers, and childcare professionals. Primary carers typically receive ongoing training by staff, including training on developing communication and play skills, and managing challenging behaviour and transitions. Fidelity measures are rarely used in standard community EI.

### Measures

The following measures were available on all children at pre-intervention and used to achieve group matching.

#### Autism Symptom Severity

The Autism Diagnostic Observation Schedule, Generic (ADOS-G; Lord et al., 2000) was used to verify diagnoses. This semi-structured, standardised measure of autism symptoms has sound psychometric properties (Lord et al., 2002). The calibrated severity algorithms introduced by Gotham et al. (2007), yielded an overall severity score. Clinicians that completed the ADOS were trained to research reliability on the administration, scoring, and interpretation of the ADOS by an accredited trainer.

#### Cognitive Functioning

The Mullen Scales of Early Learning (MSEL; Mullen, 1995) which is commonly used in children with neurodevelopmental concerns aged 0 to 5 years, eight months was used to measure cognition. Four scales were administered: receptive language (RL), expressive language (EL), fine motor (FM), and visual reception (VR). Developmental quotient scores (DQ: age equivalent scores /chronological age X 100) were derived from each subscale yielding an Age Equivalent score to assist in matching the intervention and comparison groups at pre-intervention. An average of the RL and EL DQs yield the verbal developmental quotient (VDQ), while the FM and VR DQ scores are averaged yielding the non-verbal developmental quotient (NVDQ).

#### School Age Follow-Up Measures

In addition to the ADOS, the following measures were administered to participants at the School Age Follow-Up.

#### Adaptive Behaviour

Adaptive behaviour was measured using the Parent/Caregiver Rating Form of the Vineland Adaptive Behaviour Scales, Second Edition (VABS-II; Sparrow et al., 2005). This questionnaire has good psychometric properties and was used to measure adaptive behaviour across three domains: Communication, Socialization, and Daily Living Skills (DLS). A standardized overall Adaptive Behaviour Composite (ABC) was calculated from the Communication, Socialization, and DLS domains.

#### Social Skills and Problem Behaviour

The Social Skills Improvement System Rating Scales (SSIS-RS; Gresham & Elliot, 2008) measured social skills and problem behaviours. The Parent Form of the SSIS which has adequate reliability and validity (Gresham et al., 2011) was used in the current study and is suitable for children aged three to 18 years of age, including children with autism (Clark et al., 2019; Payne et al., 2020). The social skills composite takes into account seven social behaviour domains: communication, cooperation, assertion, responsibility, empathy, engagement, and self-control. The problem behaviour composite examines bulling, hyperactivity/inattention, internalizing, externalizing, and autism spectrum behaviours. The overall standardized scores for social skills and problem behaviours were utilized here. Each domain score has a mean of 100 and standard deviation of 15. Children that scored more than one standard deviation above the mean scored within the *above average* range, with scores more than one standard deviation below the mean categorised as *below average*.

#### Play Behaviour

Play behaviour was measured with the Penn Interactive Peer Play Scale (PIPPS; Fantuzzo & Hampton, 2000). This 32-item rating scale assesses capabilities in play and can differentiate children with positive peer relationship skills from those with peer interaction difficulties. The parent version of the measure was used to assess three dimensions of play: play interaction, play disruption, and play disconnection. Play interaction captures play strengths such as helping other children, creativity, and encouraging others in play. Play disruption highlights aggressive and anti-social play behaviours while play disconnection reveals withdrawal or lack of participation in peer play. T-scores (population *M* = 50, *SD* = 10) are provided for each dimension.

#### Attitude Towards School

The School Liking and Avoidance Questionnaire (SLAQ; Birch & Ladd, 1997) captured attitudes towards school. The caregiver version of the questionnaire has two subscales: school liking and school avoidance, with higher scores indicating higher levels on the relevant dimension. The school liking subscale comprises items that measure positive attitudes towards school such as “when you wake up in the morning are you happy about going to school?”, “are you happy when you’re at school?” and “is school a fun place to be?”. Contrastingly, the school avoidance domain comprises items that tap into negative attitudes towards school such as “do you ask your mum or dad to let you stay home from school?” and “do you feel happier when it is time to go home from school?”.

### Procedure

The study protocol was granted ethics approval by La Trobe University Human Ethics Committee (approval number: UHEC-14-058). Assessments of cognitive functioning and autism symptoms conducted at baseline and follow-up were used to match participants. The school age follow-up (T2) assessments were undertaken by different clinicians who had not previously assessed the children at the earlier time points.

Of interest in this study were the caregiver questionnaires on their child’s adaptive functioning, social skills and problem behaviours, play behaviours and attitudes towards school. Caregivers either completed the questionnaires during the child’s follow-up assessment session or in their own time at home and returned in a reply-paid envelope. Due to variation in the number of caregiver questionnaires returned, data from slightly different sample sizes were available for the analyses, presented below.

## Results

The data were first screened to ensure the assumptions for MANOVA were met. Data were normally distributed and there was no evidence of multicollinearity amongst variables. As the sample sizes were uneven, Levene’s test of homogeneity of variance revealed equal population variance.

### Adaptive Behaviour

A one-way between groups multivariate analysis of variance (MANOVA) was conducted to compare groups on the three subdomains of the VABS: Communication, Socialization and DLS (see Table 2). Although children in the G-ESDM group had consistently higher mean scores at school-age (see Table 2), the groups did not differ significantly on these variables *F*(2,40) = 1.36, *p* = 0.582; *Partial Eta Squared* = 0.06).

**Table 2:** Participant adaptive behaviour, communication, socialization, and daily living skills at follow-up

	ESDM *N* = 27		Comparison *N* = 18		Group comparison		
	*M* (SD)	Descriptive range	*M* (SD)	Descriptive range	*F*	*p*	*ES*
Communication	77.78 (22.04)	Moderately low	72.56 (14.44)	Moderately low	1.36	.58	.06
Socialization	76.37 (18.87)	Moderately low	70.78 (11.92)	Very low			
Daily Living Skills	80.04 (19.37)	Adequate	73.44 (11.20)	Moderately LOW			

Descriptive range: Children’s scores relative to population norms

*ESDM* early start Denver model

*p < .05

**p < .01

^†^*p* = .05

### Social Skills and Problem Behaviour

A one-way between-groups multivariate analysis of variance (MANOVA) was conducted to compare groups on social skills and problem behaviours as measured on the SSIS. As shown in Table 3, although children in the G-ESDM group had slightly higher mean scores on social skill and lower scores on problem behaviours, the groups were not significantly different on these variables (*F* (2,40) = 1.96, *p* = 0.154; *Partial Eta Squared* = 0.09).

**Table 3:** Participant social skills and problem behaviours at follow-up

	ESDM *N* = 25		Comparison *N* = 18		Group comparison		
	*M* (SD)	Descriptive range	*M* (SD)	Descriptive range	*F*	*p*	*ES*
Social skills	71.44 (19.38)	Below average	69.89 (17.37)	Below Average	1.96	.15	.09
Problem behaviour	115.16 (12.92)	Above average	124.33 (17.42)	Above Average			

Descriptive range: Children’s scores relative to population norms

*ESDM* early start Denver model

*p < .05

**p < .01

^†^*p* = .05

### Play Behaviour

A significant multivariate group effect was found on the PIPPS (*F* (3,42) = 3.92, *p* = 0.015, Partial Eta Squared = 0.22). The univariate analyses, reported in Table 4, indicate that children in the comparison group had significantly higher PIPPS *play disruption* scores at follow-up compared to the G-ESDM group (*p* = 0.005), with a large effect size (*Partial Eta Squared* = 0.17). The univariate analyses revealed no differences between groups on the *play interaction* or *play disconnection* sub-scales.

**Table 4:** Participant play interaction, play disruption, and play disconnection behaviours at follow-up

	ESDM *N* = 25		Comparison *N* = 21		Group comparison		
	*M*	SD	*M*	SD	*F*	*p*	ES
Play interaction	28.68	2.95	34.81	3.22	1.97	.17	.04
Play disruption	48.40	1.91	56.81	2.09	8.84	.005	.17
Play disconnection	65.64	1.09	65.38	1.19	.03	.82	.00

*ESDM* early start Denver model

### Attitude Towards School

Children’s school liking and school avoidance scores are shown in Table 5. Despite higher mean school liking and lower school avoidance scores in the G-ESDM group, there was no multivariate group effect on these combined independent variables (*F* (2,37) = 2.21, *p* = 0.124; *Partial Eta Squared* = 0.11). Autistic children in the G-ESDM and comparison groups were reported to have similar attitudes towards school.

**Table 5:** Participant school liking and school avoidance at follow-up

	ESDM *N* = 22		Comparison *N* = 18		Group comparison		
	*M*	SD	*M*	SD	*F*	*p*	ES
School liking	17.91	.92	15.78	1.02	2.21	.12	.11
School avoidance	7.96	1.06	11.17	1.17			

*ESDM* early start Denver model

## Discussion

This is the first study to compare the school-aged outcomes of autistic children who had received community-based G-ESDM to those in receipt of standard community EI. The current study extends on the work of Vinen et al. (2018) that found improvements in cognitive functioning in both groups (ESDM and comparison) of children studied here irrespective of intervention type. The same 59 children participated in the current study, with a focus on their social, peer play and behavioural outcomes and attitudes towards school. While the two groups did not differ on adaptive behaviour as hypothesized, their social and peer play skills, problem behaviours, and attitudes towards school also failed to differentiate the groups, counter to the stated hypotheses. Further, groups did not differ in their peer play skills (play interaction and play disconnection) with one exception, children in the G-ESDM group displayed less play disruption with their peers than those in the comparison group according to their parents. The difficulties in adaptive and social functioning at school age is not completely unexpected as EI is known to target improvements in communication, language and cognition which have also been identified in this same cohort (Vinen et al., 2018). In the current study, the mean adaptive behaviour scores indicate that children in both groups scored in the ‘moderately low’ range, demonstrating pervasive difficulties in adaptive functioning at school age, irrespective of the EI received. Studies have also reported enduring difficulties with adaptive functioning throughout the course of childhood (Cohen et al., 2006; Eikeseth et al., 2007; Estes et al., 2015; Magiati et al., 2011; O’Connor & Healy, 2010). Further to this, the lack of difference between the groups in adaptive behaviour align with that reported by Vivanti et al. (2014), where no differences in adaptive behaviour were found immediately post intervention between children in receipt of G-ESDM and a comparison group (other community interventions). They contrast, however, with the findings of Estes et al. (2015) who reported significant improvements in adaptive functioning at follow-up in children that received ESDM, further re-iterating the variability in adaptive functioning outcomes of autistic children.

Children in both the G-ESDM and comparison groups had similar social skills and problem behaviours at school age. On the basis of parent report, children displayed fewer prosocial skills than would be expected relative to their TD peers. In turn, they showed higher levels of problems behaviours. Importantly, as autism spectrum behaviours were measured within the problem behaviours scale, it is not surprising that there is an elevation of these behaviours. Certainly, research has indicated lower levels of social skills amongst autistic children at school age, despite the provision of EI (O'Connor & Healy, 2010; Towle, et al., 2014).

In keeping with similar social skills, both groups also displayed similar levels of play interaction and play disconnection. However, children in the G-ESDM group demonstrated less play disruption, such as aggressive or antisocial behaviour, when compared to the comparison group. The significantly lower levels of play disruption may be explained by the play-based learning approach of the G-ESDM that targets children’s learning, communication and social goals while engaging them in play (Vivanti et al., 2014). While these findings are encouraging, few studies have explored these same three dimensions of play, and replication is needed to examine changes in play behaviours including play disruption, from pre-intervention to school age. Despite this positive finding, some play difficulties were also identified in the current study with both groups showing, on average, less interaction and greater disconnection in peer play compared to the standardized means. These findings partially align with the work of Clark et al. (2019) who also measured the same dimensions of play using the PIPPS in school-aged autistic children (aged 7 to 9 years). Clark et al. found that autistic children scored highest in play disconnection, experiencing the most difficulty connecting with peers, joining in and remaining engaged in play even when invited to do so by their peers. Collectively, these findings indicate that school-aged autistic children are presenting with behaviours that are counterintuitive to positive peer engagement in play, with either high levels of disruptive behaviour and/or disconnection from peers. Consequently, such known difficulties (increased disruption and disconnection in play) coupled with poor social skills, may impact children’s opportunities to develop and maintain peer relationships, another area deserving further investigation (Bauminger & Kasari, 2000).

Similarly overlooked in the EI outcome literature is children’s attitude towards school. Once again, comparable displays of school liking and avoidance behaviours were reported for children in G-ESDM and comparison groups. Encouragingly, parents/carers typically reported greater school liking than avoidance behaviours irrespective of the EI received. This is of particular interest given common reports of school refusal behaviours amongst autistic children (Bitsika et al., 2021; McClemont et al., 2020; Ochi et al., 2020) and the increased risk of school avoidance of autistic children relative to TD children (Munkhaugen et al., 2017). However, as the SLAQ (Birch & Ladd, 1997) is not a standardized measure, no further inferences can be drawn from these data. Further examination of such attitudes is warranted in future research, as behaviour associated with early school avoidance may be a risk factor for later school refusal behaviours which can subsequently impact on children’s social and academic developmental and later outcomes.

Together, the current findings demonstrate similar early school-aged outcomes amongst children who received different models of community based EI during their preschool years. Importantly, the community interventions provided to children in the treatment as usual group incorporated strategies from a number of evidence-based interventions. Even so, as indicated in the literature to date (Starr et al., 2016), despite the known positive effects of EI on the developmental trajectory of young autistic children, the difficulties facing many of these children as they enter their schooling years remain present. Adding to the current research evidence, this study identified ongoing challenges in adaptive behaviour, social skills, and play behaviour in the early school years. Thus, further supports and interventions are needed at school-age to assist autistic children to continue to develop and learn.

## Limitations

A limitation in the present study is the variability in interventions received by the participants in the comparison group. Moreover, it is not possible to comment on the fidelity of the intervention delivered as there is likely individual variability in the approaches across service providers and allied health professionals in the community. Further, the EI received relied on retrospective recall by parents and thus, may be limited by recall bias. Based on this qualitative information provided by parents at the school-age follow-up, most children received a variety of EI types, provided in an autism specific intervention service or otherwise. There was also likely variation in the resources children received when at school to assist with their learning, but these data were not systematically recorded. The lack of significant difference in children’s attitudes towards school—despite a medium effect size—may be due to an underpowered sample. Thus, further exploration of the attitudes towards school of autistic children is warranted within a larger sample.

It is important to consider the impact of situational specificity which posits that children’s behaviour varies depending on the context (Achenbach et al., 1987). This is particularly important to consider when interpreting findings that directly relate to other contexts including attitudes towards school and engagement with peers which relate to the child’s behaviours outside of their familiar home environment. For example, Clark et al. (2019) reiterates the value of multi-informant perspectives whereby each respondent provides an inherently different perspective based on experience. Despite efforts to obtain parent *and* teacher reports, only parent data could be reported due to low response rates from teachers. Thus, despite the children being of school-age, information from the school context was not available which would have been valuable in interpreting the study findings. Unlike teachers who are present in the school environment and able to observe children’s peer interactions, it is difficult for parents to report on their child’s social experiences at school, as parents are not privy to the social interactions that occur in the school setting. Therefore, when commenting on peer interactions and play, parents were asked to report any interactions they had observed between their child and other children/siblings in the community. As such, the information provided by parents needs to be considered within the context of several factors such as the child’s opportunity to engage with other children, the presence of near age siblings or cousins within the family and whether there are multiple autistic children in the family unit. The addition of an observational measure of play skills would supplement parental report providing a more in-depth picture of children’s social functioning within the context of an unstructured peer interaction in the school setting. Further to this, it is unclear how the school environment shapes children’s positive and negative behaviours which proved beyond the scope of this study. While investigations into children’s attitudes towards school is an important first step in gauging a child’s experience within their school environment, further work is needed to understand how a child on the autism spectrum interacts within their classroom and playground environments, and the subsequent impact on behaviour, learning and peer interactions. With new evidence suggesting that sensory aspects of the physical environment (noise and lighting) can impact the learning and engagement of students on the autism spectrum (Dargue et al., 2021) and that sensory difficulties are a leading barrier to participation (Ismael et al., 2018), the impacts of the environment may extend beyond engagement with learning, potentially impacting behaviour and social outcomes of autistic students.

Parents provide reliable valid reports on their child’s skills and abilities and are often used as a proxy to report on their child’s feelings (Achenbach et al., 1987; Clark et al., 2019). Yet, it is children themselves who are arguably best equipped to comment on internal, subjective experiences and attitudes (Egilson et al., 2017). However, as the current study relied on single reports from parents to capture attitudes towards school, the current study reflects parents’ *perception* of their child’s attitudes towards school, based on information communicated to them by their child, the child’s teacher, or from their own observations. To that end, research is beginning to provide adults (Teti et al., 2016) and autistic children a much-needed voice in the literature (Clark & Adams, 2020; Goodwin, 2019). The past decade has seen a push within autism research to increase the uptake of self-report methodology, to include the lived experiences of autistic individuals who are able to provide valid and reliable accounts of their *own* perceptions and experiences (Shipman et al., 2010). Importantly, future research exploring the attitudes and experiences of autistic children should seek to engage the children themselves directly in the research process, rather than relying solely on parent report.

## Conclusions

Autistic children in receipt of community-based EI continue to display moderate to low levels of adaptive behaviour, social skills, and peer play behaviours as they begin their schooling years. Children who received community-based G-ESDM intervention were indistinguishable from those who received standard community provisions for ASD on their adaptive behaviours, social skills, attitude towards school and problem behaviours at school age. However, the level of play disruption was found to be significantly higher amongst the comparison children, despite no differences in play interaction or disconnection at this age.

Overall, the school-aged autistic children studied here experienced continued difficulties despite receipt of EI during their preschool years, emphasizing the need for ongoing supports at school. It is important to consider the current findings in the context of several unknown factors which may have impacted the outcomes of children in this study. Further replication is needed to confirm the current school-aged outcomes of children following cessation of EI.
